# Autoantibodies Against Factor B and Factor H Without Pathogenic Effects in a Patient with Immune Complex-Mediated Membranoproliferative Glomerulonephritis

**DOI:** 10.3390/biomedicines13030648

**Published:** 2025-03-06

**Authors:** Alexandra T. Matola, Dorottya Csuka, Ágnes Szilágyi, Michael Rudnicki, Zoltán Prohászka, Mihály Józsi, Barbara Uzonyi

**Affiliations:** 1 Department of Immunology, ELTE Eötvös Loránd University, H-1117 Budapest, Hungary; 2 HUN-REN-ELTE Complement Research Group, Hungarian Research Network, H-1117 Budapest, Hungary; 3 Department of Internal Medicine and Hematology, Semmelweis University, H-1088 Budapest, Hungary; 4 HUN-REN-SE Research Group for Immunology and Hematology, Department of Internal Medicine and Hematology, Semmelweis University, H-1088 Budapest, Hungary; 5 Department of Internal Medicine IV, Medical University of Innsbruck, A-6020 Innsbruck, Austria

**Keywords:** complement, autoantibodies, membranoproliferative glomerulonephritis, factor B, factor H

## Abstract

**Background**: Membranoproliferative glomerulonephritis (MPGN) is an umbrella term for chronic disorders affecting the glomeruli. MPGN is often accompanied by the presence of autoantibodies against complement components. However, the actual pathogenic effects of such autoantibodies, if any, are rarely studied. In this work, we investigated the role of anti-complement autoantibodies in an IC-MPGN patient. **Methods**: The presence of autoantibodies, their binding site, isotype, and titer were analyzed in ELISA. Antibody–antigen complexes were detected in the patient’s serum using Western blot. Autoantibodies were studied in functional assays to analyze their effects on C3 convertase, complement deposition, cofactor activity, C3b binding, and hemolysis. **Results**: We identified autoantibodies against factor B (FB) and factor H (FH) in the patient’s serum. Both FB-, and FH-autoantibodies were of IgG2, IgG3, IgG4, and IgGκ, IgGλ isotypes. FB-autoantibodies bound to the Ba and the enzymatically active Bb part of FB. FH-autoantibodies bound to the N- and C-termini of FH and cross-reacted with FHL-1 and FHR-1 proteins. In vivo formed complexes of the autoantibodies with both FB and FH were detected in the IgG fraction isolated from the serum. The autoantibodies did not influence solid-phase C3 convertase assembly and its FH-mediated decay. The free autoantibodies had no effect on complement deposition and on FH cofactor activity but slightly reduced C3b binding to FH. The IgG fraction of the patient dose-dependently inhibited complement-mediated rabbit red blood cell lysis, and the free autoantibodies decreased the solid phase C3 convertase activity. **Conclusions**: This case highlights that FB- and FH-autoantibodies are not necessarily pathogenic in IC-MPGN.

## 1. Introduction

Membranoproliferative glomerulonephritis (MPGN) is a general pattern of injury in the kidney and can be further subclassified according to the pathological alterations in the glomeruli. Traditionally, the diagnosis is based on complement parameters and analysis of the biopsies by electron microscopy, immunofluorescence microscopy (IF), or immunohistochemistry. Nowadays, classification relies mostly on the findings of IF analysis [1,2,3], and serological complement assays help confirm the diagnosis [4]. Thickening of glomerular capillary, enlarged and lobular glomeruli, hypercellularity in the mesangium, endocapillary proliferation, immune complex and/or complement deposition are among the characteristic changes that can be observed in MPGN [2,5]. Chronic infections (e.g., hepatitis B and C) often cause MPGN due to antigenemia and immune complex formation and deposition in the glomeruli [6]. In the last years, evidence of glomerulonephritis as an extraintestinal complication of inflammatory bowel disease has also arisen [7].

**Figure 1:**
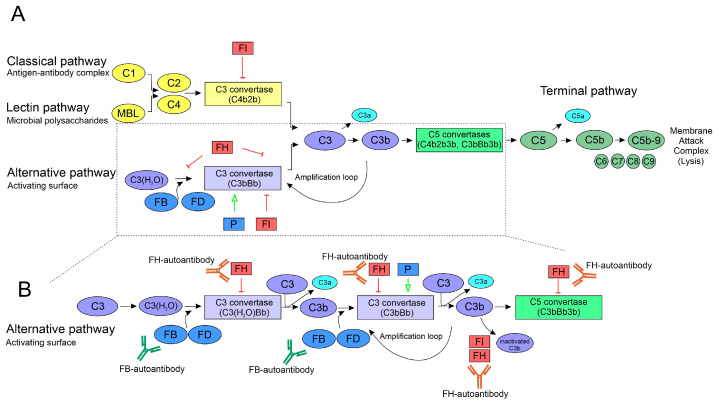
Schematic view of complement activation. (**A**) Activation of the complement system via the classical, lectin and alternative pathways, all joining at the central C3 component and leading to the common terminal pathway activation. Complement can be activated via the classical pathway (CP) by immune complexes, the lectin pathway (LP) by microbial polysaccharides, or the alternative pathway (AP) that is constantly active due to the so-called “tick-over” mechanism. Any activator stimulus leads to the formation of a convertase enzyme complex (C4b2b in the CP and LP and C3bBb in the AP) that cleaves the central C3 molecule and propagates the formation of C5 convertases. These, in turn, cleave C5 into C5a and C5b and thus bring forth the cascade to the common terminal pathway, resulting in the formation of the C5b-9 membrane attack complex. The generated anaphylatoxins C3a and C5a are important inflammatory mediators. (**B**) A more detailed delineation of alternative pathway activation depicting potential intervention points of the analyzed FB- and FH-autoantibodies. At a low rate, the reactive thioester group of C3 hydrolyses and the generated C3(H_2_O) binds factor B (FB), which, after cleavage by factor D (FD), forms the initial fluid phase C3 convertase of the AP (C3(H_2_O)Bb). It cleaves C3 into C3a and C3b, the latter being able to bind to all kinds of surfaces. If not inactivated by regulators, e.g., cell surface regulators (not depicted) or factor H (FH) and factor I (FI), C3b serves as a binding partner for FB, and after cleavage by FD, they form the solid phase AP C3 convertase C3bBb, which can be stabilized by properdin (P). The C3bBb convertase cleaves further C3 molecules that initiate new C3 convertases by binding FB, thereby amplifying AP activity (amplification loop) or transforming the C3 convertase into C5 convertase (C3bBbC3b). FH, as a main regulator, can prevent convertase formation by inhibiting FB binding to C3b, force the decay of existing convertases by displacing Bb, and assist as a cofactor in the inactivation of C3b by FI. Autoantibodies against the convertases (nephritic factors, not depicted) can stabilize the convertases and thus prolong complement activation and increase anaphylatoxin generation. Autoantibodies against FB and FH can enhance or impair the above-mentioned functions, thereby shifting the balance of complement activation and regulation.

Complement activation is a hallmark of MPGN, its subclasses showing characteristic changes based on the involvement of the various complement pathways, and the measured complement parameters are used as diagnostic markers. Complement is a complex network of pattern recognition molecules, zymogens and other components that can be quickly activated in a cascade-like manner, regulators, and cellular receptors. It serves as a first-line defense against infections and participates in the removal of dead cells and immune complexes (Figure 1) [8]. Several gene duplications, deletions, and mutations in complement genes are associated with MPGN, and autoantibodies directed against complement proteins or complexes can disturb the balance of complement activation and regulation, contributing to or leading to kidney diseases [9,10,11,12,13]. MPGN can be divided into immune complex-mediated MPGN (IC-MPGN) and C3 glomerulopathy (C3G). The latter is further classified as dense deposit disease (DDD) and C3 glomerulonephritis (C3GN), determined by the presence or absence of intramembranous dense deposits [5,14]. IC-MPGN is driven by the presence of circulating and deposited immune complexes, which result in the activation of the classical complement pathway (CP) and subsequent C3 deposition in the kidney [2,14]. Autoantibodies stabilizing the CP C3 convertase (C4b2b) called C4 nephritic factors (C4Nefs) contribute to the overactivation of complement via prolongation of the half-life of C4b2b [15,16]. However, complement alternative pathway (AP) overactivation also occurs in IC-MPGN [17].

Factor B (FB) circulates as a proenzyme in the plasma, and upon binding to C3(H_2_O) or C3b and when cleaved by Factor D (FD), its enzymatically active Bb fragment becomes part of the AP C3 and C5 convertases (C3(H_2_O)Bb, C3bBb and C3bBb3b, respectively) [18].

One of the main regulators of AP is factor H (FH), which acts at the level of the central C3 molecule. FH exhibits several complement regulatory functions: it is a cofactor for factor I (FI) in C3b cleavage, prevents convertase formation by competing with FB for C3b binding, and accelerates the decay of AP convertases [19,20]. The FH protein family consists of FH and FHL-1 (FH-like protein-1), both encoded by the *CFH* gene, and five FHR (FH-related) proteins encoded by the *CFHR* genes. All of them are composed of complement control protein (CCP) domains and the homologous domains of FH and FHRs share various degrees of amino acid sequence identity (see also Supplementary Figure S3C insert). FH consists of 20 CCPs from which the N-terminal CCPs 1-4 exhibit the regulatory functions, while the C-terminal CCPs 19-20 harbor the main ligand and surface binding sites [20]. Insufficient complement regulation might lead to pathologic overactivation. Pathogenic variants and copy number variations of the *CFH* and *CFHR* genes were described in both IC-MPGN and C3G [13].

FH-autoantibodies were also described in MPGN patients, usually with a low titer, with predominantly N-terminal binding sites and various functional effects if determined, ranging from clearly pathogenic role to mild impairment of FH functions [21,22,23,24,25,26]. By contrast, FH-autoantibodies in patients with atypical hemolytic uremic syndrome (aHUS) [27] bind to the FH C terminus, and data support a pathogenic role of aHUS-associated FH-autoantibodies [28,29,30,31,32]. Convertases and components of the convertases, i.e., C3b and FB, can also be targets of autoantibodies [15,16,33,34,35,36]. Autoantibodies directed against C3 and C5 convertases, termed nephritic factors (C3Nefs and C5Nefs, respectively) since they are associated with rare kidney disorders, stabilize the AP C3 convertase and the AP/CP/lectin pathway (LP) C5 convertases, respectively, thus leading to enhanced C3 and/or C5 cleavage and persistent complement activation [37]. All types of Nefs were described in IC-MPGN cohorts with various frequencies [15,16,33,38].

FB-autoantibodies and C3b-autoantibodies in MPGN were reported to have similar effects to C3Nefs on the complement activation by binding the C3 convertase and resulting in enhanced assembly, increased stability, and altered convertase complex regulation [34,36]. However, autoantibodies to complement components described in other diseases are not necessarily pathogenic. FH autoantibodies in non-small cell lung cancer patients proved to be protective [39], and FB autoantibodies in rheumatoid arthritis patients rather inhibited complement activation [40]. In addition, more than one type of complement-autoantibody may be concomitantly present in patients.

This study’s aim was to characterize anti-complement autoantibodies in a previously described IC-MPGN patient [41]. By detailed functional analysis, we demonstrate that such antibodies in MPGN are not necessarily pathogenic.

## 2. Materials and Methods

### 2.1. Serum Samples

In this study, a serum sample from an IC-MPGN patient was analyzed. Normal human serum was collected from healthy individuals after informed consent in accordance with the Declaration of Helsinki. Samples were collected between 2012 and 2018. Oral consent was obtained, and the measurements were performed using samples obtained for diagnostic purposes. The patient (HUN593) was previously described as part of a registry [41]. Medical data were accessed for research purposes on 5 October 2024. The authors had no access to information that could identify individual participants during or after data collection. The study was approved by the National Ethical Committee (8361-1/2011-EKU).

### 2.2. Proteins, Antibodies, and Red Blood Cells

Sources of commercially obtained materials are listed in Supplementary Table S1. IgG fractions were isolated from the serum samples using HiTrap Protein G HP column (Cytiva, Marlborough, MA, USA). IgG-FH and IgG-FB immune complexes were removed prior to functional assays by HiTrap NHS-activated HP columns (Cytiva) coupled with anti-FH or anti-FB IgGs isolated from goat antisera (termed complex-free IgG). Recombinant FH fragments consisting of CCP1-4, CCP8-14, CCP15-20, CCP19-20, as well as FHL-1 and FHR-1, were produced in *Spodoptera frugiperda* (Sf9) insect cells (#600100; Oxford Expression Technologies Ltd., Oxford, UK) using baculovirus expression system. Proteins were purified from the cell culture supernatant by nickel affinity chromatography.

### 2.3. Microtiter Plate Assays

Autoantibodies against complement components in the serum of the IC-MPGN patient were detected in ELISA. Diluted in DPBS and immobilized on microtiter plates were 2 µg/mL C1q, 5 µg/mL FH, FB, C3b, and the negative controls HSA or alpha-1 antitrypsin (A1AT). Wells were blocked with 5% BSA in DPBS-0.1% Tween-20, then patient-derived and healthy serum samples were added at a dilution of 1:50. The solid phase C3 convertase, C3bBbP was built up using purified components on microtiter plates (see Section 2.7), then serum samples diluted 1:50 in DPBS were added to the wells. For the detection of C1q-autoantibodies DPBS, 1 M NaCl was used to avoid non-specific binding. Autoantibodies bound from the serum were detected with HRP-labelled goat anti-human IgG, anti-human IgA, or goat anti-IgM antibodies diluted at 1:1000, using high-sensitivity TMB substrate solution (BioLegend, San Diego, CA, USA). The absorbance of the samples was measured at 450 nm and at the reference wavelength of 620 nm. We considered the patient autoantibody-positive if the mean A450 signal on the immobilized complement proteins was more than 2-fold higher than on HSA or A1AT. For isotype determination, isolated patient and control IgG were incubated on the FH, FB, and negative control protein, and isotype-specific mouse monoclonal antibodies at 1:250 dilution, HRP-labelled mouse anti-IgG4 at 1:500 dilution and HRP-labelled goat anti-mouse Ig at 1:1000 dilution were used.

Binding sites of the FH autoantibodies were determined using recombinant FH fragments, FHL-1, and FHR-1. Full-length FH and FH fragments CCP1-4, CCP8-14, CCP15-20, and CCP19-20, as well as FHL-1 and FHR-1, were immobilized in microplate wells. IgG fraction of the patient and healthy control were added, and the bound IgG was detected with 1:1000 diluted HRP-conjugated anti-human IgG. To analyze the binding sites of the FB-autoantibody, Ba and Bb fragments were immobilized in microplate wells and, after blocking, incubated with IgG of the patient or healthy control at 200 µg/mL. The bound IgG was detected with HRP-conjugated goat anti-human IgG diluted at 1:1000. Ba- and Bb-specific monoclonal antibodies were used to inhibit the autoantibody binding to FB. After blocking, the immobilized FB was preincubated with 10 µg/mL of the monoclonal antibodies, and then binding of the patient IgG was detected with HRP-conjugated anti-human IgG at 1:1000 dilution. Autoantibody titer was determined in ELISA with serial dilutions of serum starting from 1:50 on the immobilized complement proteins.

The binding of C3b to FH, FH15-20 fragment, FHL-1, and FHR-1 in the presence of IgG from the patient was investigated in ELISA. FH, FH15-20, FHL-1, and FHR-1 were immobilized at 5 µg/mL and, after washing and blocking with 5% BSA in DPBS containing 0.1% Tween-20, preincubated with 500 µg/mL IgG from the patient or healthy donors for 15 min. Then, C3b was added at 5 µg/mL final concentration and incubated for another 45 min. The bound C3b was detected with rabbit anti-C3c followed by HRP-labelled swine anti-rabbit Ig, both diluted at 1:1000.

### 2.4. Complement Activation Assays

The effect of the patient’s autoantibodies on the cofactor activity of FH in the FI-mediated cleavage of C3b was investigated in fluid-phase cofactor assays. Briefly, 200 ng C3b, 100 ng FI, and 147 ng FH were incubated with or without 10 µg IgG from the patient and healthy donors for 1 h at 37 °C. Samples were separated by sodium dodecyl sulfate-polyacrylamide gel electrophoresis (SDS-PAGE) as described below for the Western blot analysis.

Fluid-phase complement activation was tested as described by Zhao et al. [42], with modifications. Briefly, 10% complement active normal human serum (QuidelOrtho, San Diego, CA, USA) was preincubated in Mg^2+^-EGTA containing buffer with complex-free IgG (see above) of the patient or healthy donors, or with 5 × 10^6^ rabbit red blood cells (RRBC) as positive control, for 15 min at 37 °C in Eppendorf tubes, then remaining complement activity was measured. To this end, microplate wells (Maxisorp, Nunc Wiesbaden, Germany) were coated with 10 µg/mL LPS and blocked with 2% BSA in DPBS. The preincubated serum samples were then added to the LPS-coated wells for 1 h at 37 °C. C3-fragment deposition was detected with HRP-conjugated goat anti-C3 diluted at 1:4000, and C5b-9 was detected with mouse anti-C5b-9 and HRP-conjugated goat anti-mouse antibodies, both diluted at 1:1000.

### 2.5. Western Blot Analysis

To analyze autoantibody–antigen complexes, 10 µg patient or control IgG was separated by 10% SDS-PAGE and transferred to a nitrocellulose membrane. The presence of FB and FH in the samples was detected with 1:5000 diluted goat anti-FB or mouse anti-FH antibody at 0.5 µg/mL dilution and 1:5000 diluted HRP-labelled rabbit anti-goat Ig or 1:2000 diluted goat anti-mouse Ig, respectively.

Samples of the fluid-phase cofactor assay were separated on 8% SDS-PAGE under reducing conditions. After transferring the proteins to the nitrocellulose membrane, C3b cleavage fragments were detected with goat anti-human C3 F(ab′)_2_ diluted at 1:2000 and HRP-labelled rabbit anti-goat Ig antibodies at 1:2000 dilution.

### 2.6. Hemolysis Assay

Incubated were 5 × 10^6^ rabbit red blood cells (RRBCs), with 7% normal human serum and serial dilutions of patient or control IgG in HEPES-Mg^2+^-EGTA buffer (20 mM HEPES, 7 mM MgCl_2_, 10 mM EGTA, 144 mM NaCl, 1% BSA, pH 7.4) for 30 min at 37 °C. Samples were centrifuged, and the absorbance of the supernatant was measured at 414 nm.

### 2.7. Solid Phase C3 Convertase Assay

Solid phase C3 convertase of the AP (C3bBbP) was generated on microtiter plates (Maxisorp, Nunc, Wiesbaden, Germany). 5 µg/mL C3b was immobilized and after washing, 2 µg/mL FB, 4 µg/mL properdin (Factor P), and 0.1 µg/mL FD were added together in DPBS containing 0.1% Tween-20, 4% BSA and 2 mM NiCl_2_ for 1 h at 37 °C. In some experiments, patient IgG at 500 µg/mL was added together with convertase components to analyze the effect on convertase formation, which was detected using goat anti-FB at 1:1000 dilution. Convertase decay was measured by incubating the convertase with IgG and detecting the remaining convertase with anti-FB. We analyzed FH-mediated convertase decay by preincubating the convertase for 15 min with 50 µg complex-free IgG before the addition of 1 µg/mL FH. After incubation, the convertase was detected with goat anti-FB antibody and HRP-labelled rabbit anti-goat Ig.

The activity of the convertase was analyzed by adding 10 µg/mL C3 for 1 h at 37 °C and measuring the amount of the cleavage product C3a from the supernatant by ELISA (Quidel)Ortho, San Diego, CA, USA.

### 2.8. Statistical Analysis

Statistical analyses were performed with GraphPad Prism version 6.01 for Windows (GraphPad Software, San Diego, CA, USA). A *p*-value ≤ 0.05 was considered statistically significant. To compare the effects of IgG isolated from the patient or healthy controls, we used one-way ANOVA with Dunnett’s multiple comparison test and Geisser-Greenhouse correction. Blot images were processed, and figures were generated with CorelDRAW version 23.1.0.389 (2021, Corel Corporation, Ottawa, ON, Canada).

## 3. Results

### 3.1. Patient Description

The 23-year-old patient was admitted from the Gastroenterology to the Nephrology department after the occurrence of two episodes of an acute kidney injury with very low C3 levels, proteinuria, and hematuria. In the previous 3 years, he was treated for Crohn disease with various medications, which showed either no effect or unbearable side effects. In the weeks before admission to the Nephrology unit, he had a highly active Crohn’s disease with diarrhea several times per day under treatment with Adalimumab 40 mg every other week and Budesonide 9 mg daily. Laboratory values were as follows: serum creatinine 1.51 mg/dL, urine protein to creatinine ratio 400 mg/g, urine albumin to creatinine ratio 177 mg/g, serum albumin 1920 mg/dL, hematuria > 250/µL, C3 7 mg/dL, C4 14.2 mg/dL. Autoimmunological diagnostics were unremarkable. Due to concomitant anemia and thrombocythemia (but no LDH elevation), screening for haptoglobin (elevated) and ADAMTS13 activity (elevated) was performed, and therefore, TTP and HUS could be ruled out. Finally, a kidney biopsy was performed, which gave the result of C3-glomerulonephritis. In the light microscopy, glomeruli showed a diffused and global basement membrane fragmentation and double contours, as well as a slight endocapillary and mesangial cell proliferation. In indirect immunohistochemistry, the most striking finding was a granular to short linear strong C3 deposition along practically all of the glomerular capillary walls. IgM was also present in the same location but to a much lesser extent. The remaining immunoglobulins and C1q did not show any pathological deposits in the glomeruli. Electron microscopy showed an immune complex glomerulonephritis with subendothelial deposits and at least partially a membranoproliferative pattern of injury. The electron microscopy picture confirmed the light microscopy diagnosis. During further visits at the clinic, kidney function normalized, and urine protein to creatinine ratio was <0.5 g/g; treatment consisted of Adalimumab, Methylprednisolone, Mycophenolate Mofetil, but also Ciprofloxacin, Rifaximin, and Metronidazole. During follow-up, proteinuria went into remission, and the patient did not show up for further examinations. A sample taken during this follow-up period was sent for complement analysis to the Research Laboratory—Department of Internal Medicine and Hematology at Semmelweis University, and it was used in this study as well.

As described earlier [41], the sample showed decreased C3 level (41; ref: 90–180 mg/dL), total complement activity (35; ref: 48–103 CH50/mL) and alternative pathway activity (16; ref: 70–105%), and increased sC5b-9 level (368; ref: 110–252 ng/mL); C4 and FH levels were in normal range. The coding region of selected disease-associated genes (*CFH*, *CFI*, *CD46*, *THBD*, *CFB,* and *C3*) was screened in the patient, but no pathogenic variant was revealed. By analyzing the *CFHR5* gene, two variants of unknown significance were identified: an insertion causing premature stop codon (c.486dup, p.E163Rfs*35) and a missense change (c.622T > C, p.C208R) that was reported previously to segregate on the same allele with the c.486dup [43], or co-occurred in the same patient without testing segregation [44,45], suggesting that the two variants can be on the same allele in our patient as well.

### 3.2. Autoantibody Screening

Antibodies reacting with FB in the IC-MPGN patient’s serum were described by Garam et al. [41]. Here, we further analyzed the serum of the patient for autoantibodies against FH, C3b, C1q, and the solid phase C3bBbP convertase. We could confirm the IgG FB-autoantibodies; furthermore, we could detect IgG FH-autoantibodies in the serum of the patient (Supplementary Figure S1A,B). Anti-FB and anti-FH IgM or IgA, anti-C1q, anti-C3b, and anti-C3bBbP IgG could not be detected (Supplementary Figure S2). Autoantibody titer was determined from serial dilutions of the patient’s or healthy control’s serum on immobilized FB and FH. Both autoantibodies have a relatively high titer of 1:3200 (Supplementary Figure S1C,D), comparable to other FB-autoantibodies found in a DDD patient [36] or other FH-autoantibodies found in MPGN [24].

### 3.3. Autoantibody Characterization

To map the binding sites of FB autoantibodies, we analyzed the autoantibody binding to the two main functional parts of FB, the ligand binding Ba and the enzymatically active Bb. The autoantibodies bound to full-length FB and Ba, and a weak binding was also detected on Bb (Supplementary Figure S3A). We also used monoclonal antibodies in inhibitory experiments to map the binding site. The FB-autoantibody binding to FB was decreased after preincubating FB with anti-Ba antibody and goat anti-FB antiserum, confirming the binding site in Ba. However, none of the tested Bb-specific monoclonal antibodies reduced the autoantibody binding (Supplementary Figure S3B).

To determine the binding sites of the FH autoantibodies, we used recombinant FH fragments covering the whole protein, as well as FHL-1 and FHR-1. The autoantibodies bound to full-length FH and with various avidity to all parts of it. We detected the strongest binding on the C-terminus (CCP15-20, CCP19-20), somewhat weaker binding at the N-terminus (CCP1-4), and a very weak binding in the middle region (CCP8-14) of FH. The autoantibodies also cross-reacted with FHL-1 and FHR-1, showing similarly strong signals as the homologous FH fragments CCPs 1-4 and CCPs 15-20, respectively (Supplementary Figure S3C).

The isotypes of FB-autoantibodies and FH-autoantibodies were determined using isotype-specific monoclonal antibodies. We found various IgG isotypes for both autoantibodies: IgG2, IgG3, IgG4, IgGκ, and IgGλ (Supplementary Figure S3D,E) were detected for both FB and FH autoantibodies.

**Figure 2:**
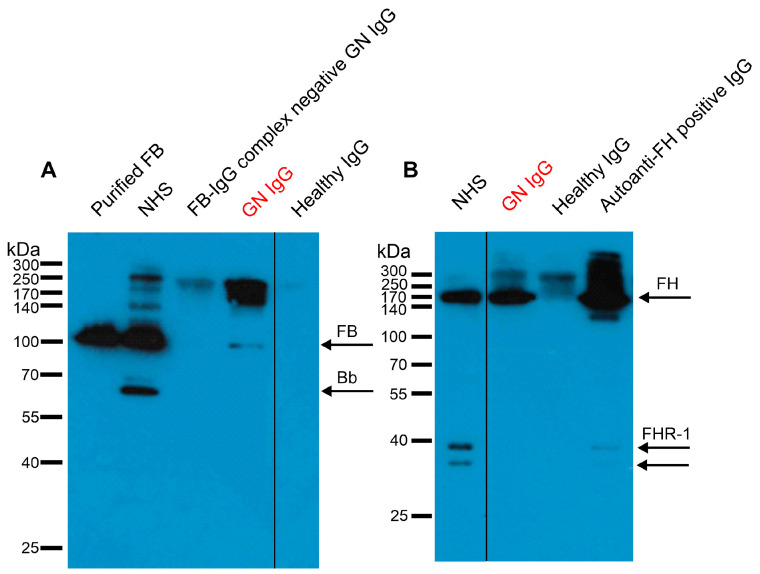
Immune complexes formed in vivo were detected in the IgG fraction of the patient. IgG isolated from the patient (indicated in red) and healthy donors were separated on SDS-PAGE, and the presence of FB (**A**) and FH (**B**) was detected in Western blot with polyclonal goat anti-FB (**A**) and monoclonal mouse anti-FH, also recognizing FHR-1 (**B**). Purified FB, IgG of a FB-IgG complex negative GN patient, normal human serum (NHS), IgG of healthy donors (**A**), and IgG of an autoanti-FH positive aHUS patient forming IgG-FH and IgG-FHR-1 complexes (**B**) were run and are shown as controls. The bands seen above FB (**A**) and FH (**B**) are probably due to the cross-reactivity of the secondary antibodies with human IgG. Blots are representative of three experiments. Original blots are presented in Supplementary Figures S4 and S5. FB: factor B, FH: factor H, FHR: factor H-related, aHUS: atypical hemolytic uremic syndrome, GN: glomerulonephritis.

Next, we wanted to investigate whether the FB- and FH-autoantibodies have high enough avidity to form complexes with their target protein in vivo. Therefore, the IgG fraction of the patient, a healthy control, and an aHUS patient, together with technical controls, were analyzed by SDS-PAGE and Western blotting. The in vivo formed autoantibody–antigen complexes fell apart during electrophoresis, and with specific antibodies, we could detect the presence of both FB and FH in the patient’s IgG (Figure 2A,B). A clear band at the size of FB in the patient’s sample (lane 4, GN IgG) points to the presence of autoanti-FB–FB complexes in the patient IgG, whereas no FB band was detected in the FB-IgG complex negative GN IgG and healthy IgG. To detect FH, we used a mouse monoclonal antibody (C18/3) that also recognizes FHR-1 (Figure 2B, lane 1). We detected a clear band at the size of FH in the patient’s IgG (lane 2) and the FH-autoantibody positive aHUS patient’s sample (lane 4) but not in the healthy control IgG (lane 3). Unlike in the aHUS IgG, FHR-1 bands were not visible in the GN patient’s IgG. Autoantibody characteristics are summarized in Table 1.

### 3.4. Effects of the Autoantibodies on FB and FH Functions

**Figure 3:**
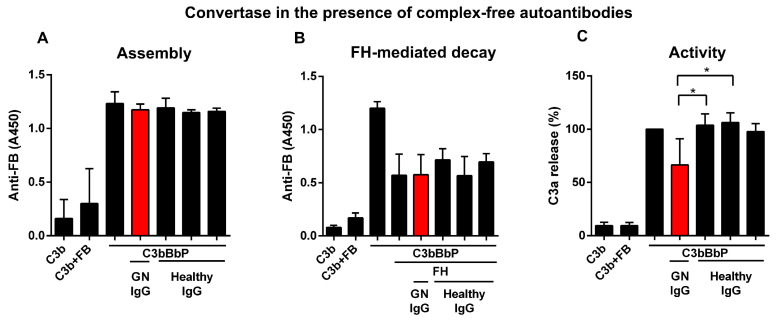
Complex-free autoantibodies reduced the activity of the solid phase C3 convertase (C3bBbP). (**A**) Patient IgG (GN IgG, shown in red) had no effect on the assembly of the solid phase C3 convertase when added together with the components (FB, FD, and properdin) of the convertase during assembly. Data are mean ± SD from three independent experiments. (**B**) Complex-free autoantibodies do not affect the FH-mediated decay when added to the convertase together with FH. Data are mean ± SD from three independent experiments. The results of two healthy controls have already been published [40]. (**C**) Assembled convertase was preincubated with IgG from the patient or healthy donors, then C3 was added and incubated at 37 °C. The generated C3a was measured in the supernatant with a specific C3a ELISA. Autoantibodies of the patient reduced the activity of the convertase. Data were normalized to the values obtained without the addition of IgG. Data are mean ± SD from four independent experiments. One-Way ANOVA, * *p* = 0.0222.

To analyze the effects of the autoantibodies, FB and FH present as part of the autoanti-FB–FB and autoanti-FH–FH complexes were removed by affinity chromatography. Since FB-autoantibodies are bound to both Ba and Bb, we measured their effect on AP convertase formation, where Ba can play a role, and on AP convertase stability and activity where the enzymatic activity of Bb is needed. In solid phase convertase assay, the convertase components FB, FD, and properdin were mixed with patient IgG or healthy control IgG and then added to the C3b-coated wells. Assembly of the convertase was detected with anti-FB antibody. We did not detect any effect of the autoantibodies on convertase formation, compared to the convertase incubated with healthy control IgGs or without IgG (Figure 3A).

Next, we investigated the effect of the patient IgG on the stability of the convertase. Following convertase assembly and preincubation with patient IgG or healthy IgG, FH was added, and decay of the convertase was measured. The detected signal for the remaining convertase was the same for the patient and control IgG and for the sample without IgG (Figure 3B), suggesting that the patient IgG could not stabilize the convertase. In further experiments, the effect of the autoantibodies on the convertase’s activity was tested. The assembled convertase was incubated with patient or healthy control IgG, then the unbound IgGs were washed away, and C3 was added to the wells. The supernatant was collected and the generated C3a was determined using a commercial EIA kit. The autoantibodies of the patient significantly decreased the activity of the solid phase C3 convertase compared to the healthy control IgGs (Figure 3C).

**Figure 4:**
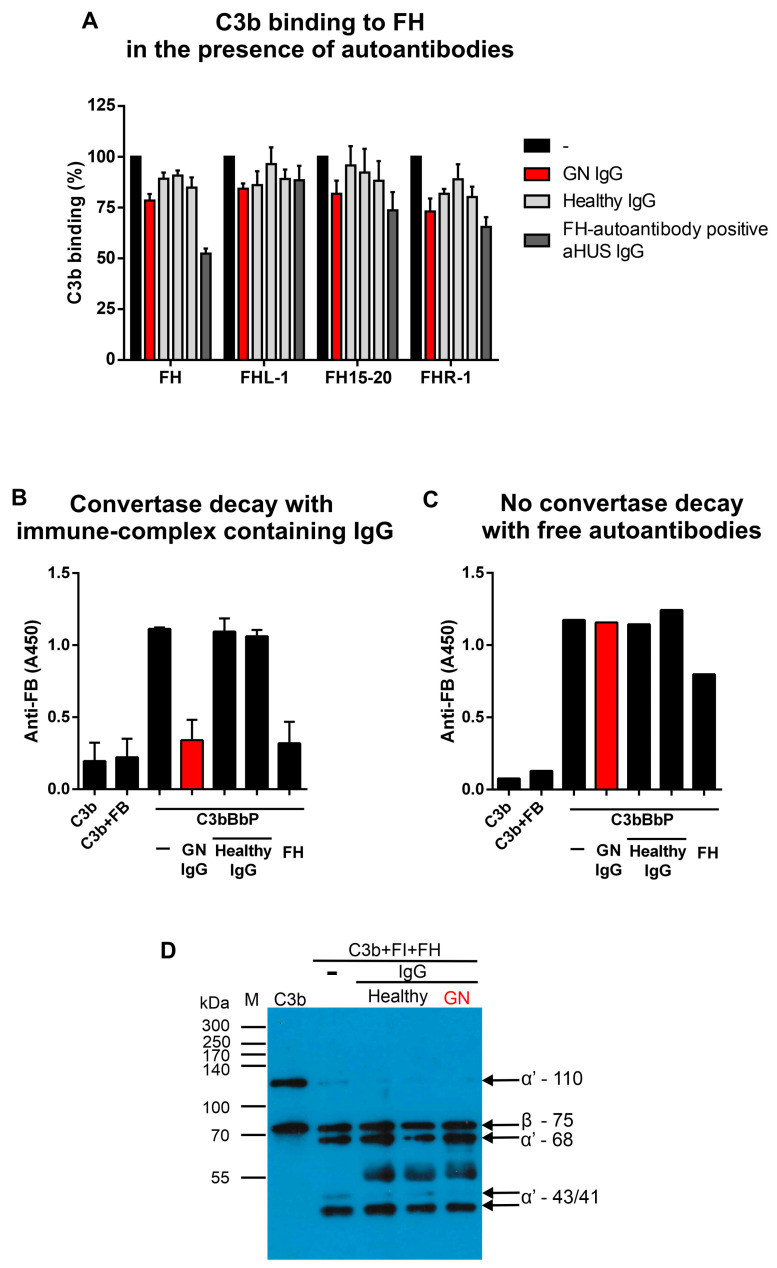
C3b binding and complement regulatory functions of FH in the presence of the autoantibodies of HUN593 patient. (**A**) C3b binding to FH was only slightly affected by the presence of free autoantibodies in the GN patient (GN IgG, shown in red). Full-length FH, its C-terminal fragment CCPs 15-20 (FH15-20), FHL-1, and FHR-1 were immobilized and preincubated with IgG of the GN patient, healthy controls, or an FH-autoantibody positive aHUS patient before adding C3b. C3b binding was normalized to the values obtained by C3b added without IgG (100%) and is mean ± SD from five independent experiments. (**B**) FH in the IgG fraction of the patient (in IgG-FH complexes) is still functional and has decay-accelerating activity. The solid phase AP convertase was assembled in microplate wells and incubated with IgG from the patient or healthy controls. The remaining convertase was detected with goat anti-FB antibody. Data are mean ± SD from two independent experiments. (**C**) Complex-free autoantibodies of the patient do not enhance convertase decay. The assembled convertase was incubated with complex-free IgGs or purified FH as control, and the remaining convertase was detected with goat anti-FB antibody. Data of a single measurement are shown. (**D**) FH retained its cofactor activity in the FI-mediated cleavage of C3b when added together with the complex-free IgG of the patient. C3b, FI, FH, and IgG were incubated at 37 °C, then C3b cleavage products were analyzed by reducing SDS-PAGE and Western blotting using goat anti-C3 and HRPO-conjugated rabbit anti-goat Ig. C3b α’ chain cleavage fragments at 68 and 43/41 kDa were detectable without IgG (lane 2), with healthy control IgGs (lanes 3 and 4), as well as with GN IgG (lane 5). The band at appr. 55 kDa in lanes 3, 4, and 5 is probably the heavy chain of the IgG due to the cross-reactivity of the secondary antibody. The blot is representative of three experiments. The original blot is presented in Supplementary Figure S6.

The effects of FH-autoantibodies were determined by measuring FH–C3b interaction, FH decay accelerating activity, and FH cofactor activity in the presence of patient IgG. Since we found both N- and C-terminally binding FH–autoantibodies, we wanted to determine which part and function of FH might be affected. To this end, we immobilized FH, FH15-20, FHL-1, and FHR-1 and preincubated with the IgGs. Then, C3b was added, and its binding was detected with a polyclonal anti-C3 antibody. In the case of full-length FH, the aHUS IgG strongly reduced C3b binding compared to the healthy control samples, whereas the GN IgG showed weaker inhibition, which was statistically not significant. When looking at the C-terminal FH15-20 fragment and FHR-1, the inhibitory effect of the aHUS and GN IgG was comparable. By contrast, C3b binding to FHL-1 was not inhibited by either patient IgG (Figure 4A). This suggests that the GN IgG might affect the surface binding capacity of FH, similar to the aHUS autoanti-FH IgG.

Convertase decay acceleration assays were performed to further analyze the FH-autoantibodies’ effect on FH functions. The isolated patient IgG containing autoanti-FH–FH complexes were added to the assembled solid phase AP convertase, and after the incubation, FB signals were measured. The IgG of healthy controls that do not contain FH had no effect on the convertase, and the signals were comparable with the buffer alone. However, patient IgG decreased the convertase signal, similar to the effect of FH alone, which was used as a control in separate wells (Figure 4B). To prove that this effect is due to FH present in complex with the autoantibodies in the IgG preparation, complex-free IgG was also used in a separate experiment. In this case, complex-free patient IgG did not induce convertase decay (Figure 4C). These results imply that FH bound in the complex by FH-autoantibodies retains its regulatory functions.

Next, we tested the autoantibodies’ effect in fluid phase cofactor assay when we determined the FI-mediated C3b cleavage in the presence of the complex-free IgG (Figure 4D). C3b was incubated with FI, FH, and IgG, and then the samples were analyzed using SDS-PAGE and Western blot. When FH is present, FI can cleave C3b, resulting in the disappearance of the 110 kDa α′ chain and the appearance of its 68, 43, and 41 kDa fragments (Figure 4D, lane 2). No difference could be seen when the IgG of healthy controls (lanes 3, 4) or the patient (lane 5) was mixed in the reaction, suggesting that the autoantibodies did not impair FH cofactor activity.

### 3.5. Effect of Patient IgG on Complement Activation

**Figure 5:**
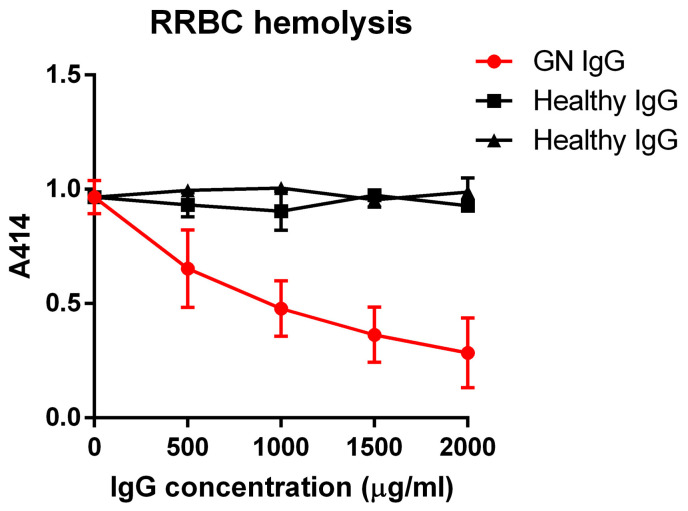
GN patient’s IgG dose-dependently inhibited complement-mediated RRBC lysis in normal human serum. Patient and healthy control IgG were mixed with complement active normal human serum, added to RRBCs, and samples were incubated at 37 °C for 30 min. Cells were pelleted by centrifugation, and the absorbance of hemoglobin in the supernatant was measured at 414 nm. Data represent means ± SD from two independent experiments. The results of the two healthy controls were already published [40]. RRBC: rabbit red blood cell.

To determine the effects of the FH- and FB-autoantibodies together on complement activation, we mixed the IgG with normal human serum and used rabbit red blood cells (RRBCs) as an activator surface and investigated fluid-phase complement activation as well. In the hemolysis assay, RRBCs were mixed with serial dilutions of the IgG isolated from the patient or healthy donors and with normal human serum. RRBCs are lysed in normal human serum. IgG from healthy controls did not affect RRBC lysis; however, the patient’s IgG dose-dependently decreased the hemolysis, probably due to the presence of FH in complex with the autoanti-FH (Figure 5).

**Figure 6:**
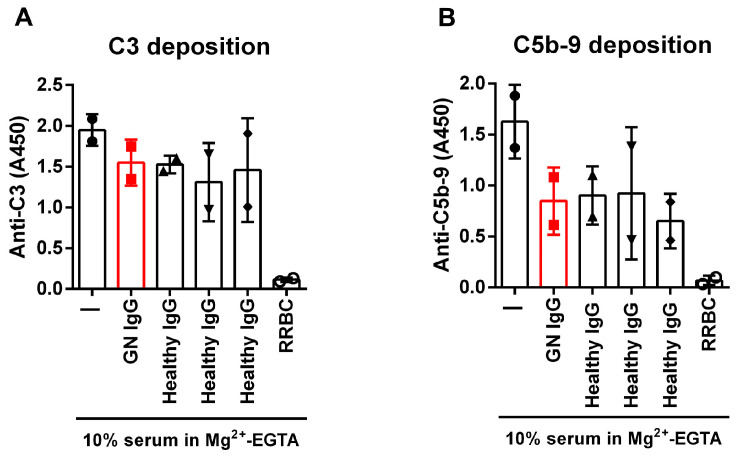
Patient IgG does not impair fluid-phase complement activation. The complex-free IgG of the patient (GN IgG, shown in red) or healthy donors was incubated with normal human serum in microtubes and then added to LPS-coated microplate wells; as a positive control, serum was preincubated with RRBCs to activate complement. The remaining complement activity was measured as C3 (**A**) or C5b-9 (**B**) deposition. The patient’s IgG had no effect on the complement activation measured as C3 (**A**) or C5b-9 (**B**) deposition compared to healthy control IgGs. Data are mean ± SD from two independent experiments. RRBC: rabbit red blood cell.

Next, we investigated whether the autoantibodies affect fluid phase convertase formation and/or complement activation. Patient and control IgGs were incubated with normal human serum in microtubes in Mg^2+^-EGTA-containing buffer; thus, CP activation by immune complexes in the IgG was inhibited. To measure the remaining AP activity, these samples were added to LPS-coated wells that induce AP activation, and then C3 (Figure 6A) and C5b-9 (Figure 6B) deposition were determined. In samples where the serum was preincubated with IgG from healthy donors, we expected no impairment in AP activation and a positive signal for C3 and C5b-9 on LPS. As a positive control, serum was incubated with RRBCs, which activate the complement; thus, due to complement consumption, reduced C3 and C5b-9 signals can be detected on the LPS-coated surface. The patient’s IgG had no effect on the complement deposition of LPS compared to the healthy IgGs (Figure 6A,B), suggesting that the autoantibodies do not affect fluid phase AP activation. The summary of the functional effects of the autoantibodies is presented in Table 1.

## 4. Discussion

In this study, we characterized IgG autoantibodies against FB and FH in a patient diagnosed with IC-MPGN who was negative for C3Nef in the traditional hemolytic assay. While the presence of FH- and FB-autoantibodies was reported in aHUS and C3GN [46,47], their function had not always been investigated in detail. In contrast to previous studies reporting autoantibodies against FB and FH that boost complement activation (presented in greater detail in the Introduction) [21,24,25,28,36], here, we report in a single patient both types of autoantibodies having a restrictive effect on complement activation by inhibiting the C3 convertase activity and not interfering with the FH-mediated complement regulation. Thus, while the exact pathomechanism leading to MPGN in the patient is unknown, these autoantibodies are unlikely to be pathogenic.

In line with the IC-MPGN diagnosis, the patient had elevated plasma level of the complement activation product sC5b-9 and decreased C3 level. Although IC-MPGN is usually characterized by Ig and C3 deposits in the kidney and classical pathway overactivation [2], this patient was reported to have the C3 level, AP50, and CH50 below the normal range, indicating AP overactivation as well. The underlying pathological processes resulting in AP overactivation are not completely understood yet, though mutations in the genes of the AP components or autoantibodies targeting these proteins were described in some cases of MPGN [21,23,24,38,48,49,50,51]. This patient does not have any mutation in the AP regulators FH, FI, or membrane cofactor protein but was tested anti-FB and anti-FH positive (Supplementary Figure S1A,B), which was suggestive of a possible, eventually combined pathologic role of these autoantibodies.

Since FB takes part in the generation of the AP C3 convertase, autoantibodies targeting FB bind to the C3 convertase as well and influence the convertase functions, having similar effects to C3Nefs. Previously reported autoantibodies bound only to the Bb part [34,36,40] and recognized their target both in uncleaved form in the fluid phase and solid phase and in cleaved form (i.e., Bb) as part of the AP C3 convertase [34]. Similarly, these FB-autoantibodies recognized intact FB as well as cleaved FB (Supplementary Figure S3A,B, and Figure 2A). We identified various binding sites and isotypes of the autoantibodies (Supplementary Figure S3), suggesting their oligoclonal origin. Although they are bound to both functional parts of the protein, Ba and Bb, they do not interfere with convertase formation or FH-mediated convertase decay (Figure 3A,B). However, in contrast to C3Nefs and FB-autoantibodies reported in kidney disease patients enhancing C3 convertase stability and/or activity [34,36,42], they did inhibit convertase activity (Figure 3C). Interestingly, similar convertase inhibitory effects were described for FB-autoantibodies reported in a rheumatoid arthritis patient [40].

FH, the main regulator of the AP, is also a common target of autoantibodies in autoimmune kidney disorders, and FH autoantibodies often prove to be pathogenic as they affect FH regulatory activity or FH binding to host surfaces that need to be protected. So far, the published FH-autoantibodies in MPGN showed binding mostly to the N-terminus of FH [23,24,25], while in aHUS, FH-autoantibodies bind predominantly to the C-terminus [28,30], reflecting a major difference in the pathological processes between the two diseases. Although the surface regulation of complement is disturbed in aHUS, complement activation is kept under control in body fluids since the regulatory domains of FH are not affected by most of the autoantibodies. In MPGN, the regulatory functions are or might be affected by FH-autoantibodies, disturbing complement regulation both in the fluid phase and on surfaces [21,23,24,25]. Unlike the previous reports of MPGN patients with FH-autoantibodies, in this IC-MPGN patient, the autoantibodies showed binding on both the N- and C-termini of FH, which together with the various isotypes identified (Supplementary Figure S3C,E) imply oligoclonal origin of the autoantibodies. Due to the high amino acid sequence similarity among the FH protein family members, the autoantibodies cross-reacted with immobilized FHL-1 and FHR-1 (Supplementary Figure S3C). Such cross-reactivity was also reported for other anti-FH autoantibodies for FHL-1 or FHR-1 but not for both proteins [25,30,52]. C-terminally binding, pathogenic FH-autoantibodies described in aHUS or potentially pathogenic FH-autoantibodies in neuromyelitis optica spectrum disorder patients impaired the FH–C3b interaction [28,30,53,54,55], whereas non-pathogenic FH-autoantibodies in lupus nephritis patients did not influence C3b binding [56] and protective autoantibodies even increased C3-deposition in cancer patients [39]. In this patient, although FH-autoantibodies bound to both the N- and C-termini of FH, C3b binding was only slightly affected by the autoantibodies (Figure 4A), but all the investigated regulatory functions of FH were retained. Under physiological conditions, FH can dissociate Bb from the formed C3 convertases to restrain complement activation. FH-autoantibodies of this patient had no effect on the FH-mediated C3 convertase decay, supporting the non-pathogenic role of these autoantibodies (Figure 3B). Moreover, the IgG fraction of the patient containing autoantibody–FH complexes was able to bring forth the decay, pointing to a preserved FH function (Figure 4B). FH plays a crucial role in the FI-mediated cleavage and inactivation of C3b. Two MPGN patients were reported with FH-autoantibodies inhibiting the cofactor activity of FH and C3b cleavage by FI [22,25], and three out of eight FH-autoantibodies from patients with AP-mediated glomerulopathies impaired, but not completely inhibited FH cofactor function [23]. The autoantibodies analyzed in the current study did not influence the C3b cleavage, also proving the undisturbed FH functionality (Figure 4D). Thus, considering earlier reported function-blocking FH-autoantibodies, the presented data imply a harmless nature of the patient’s FH-autoantibodies.

When analyzing the patient IgG as a whole, not dissecting the functions of the various autoantibodies, we found neutral or rather protective net effects. Characterization of various C3Nefs revealed both inhibitory and enhancing effects on fluid-phase complement activation [42]. In our assay, complex-free patient IgG, when added to NHS in the fluid phase, did not differ from healthy control IgGs in affecting C3 or C5b-9 deposition as a measure of remaining complement activity in the serum (Figure 6), pointing rather to a harmless nature of these autoantibodies. C3Nefs are often found to enhance sheep red blood cell (SRBC) or RRBC lysis in normal human serum due to convertase stabilization [16,57]; this is also the basis of the clinical diagnostic test [58,59]. This patient was tested negative for C3Nef, so no elevated RRBC lysis was expected. Surprisingly, patient IgG prevented RRBC lysis in a dose-dependent manner (Figure 5), proving to be protective and showing distinct effects from convertase stabilizing and thus complement activating C3Nefs and previously described FB- or FH-autoantibodies. The presence of non-pathogenic anti-complement autoantibodies with relatively low titer and of oligoclonal origin have been described in other diseases, e.g., for FH-autoantibodies in lupus nephritis [56] and in lung cancer [39,60], and for FB-autoantibodies in rheumatoid arthritis [40]. The generation of such non-pathogenic or protective autoantibodies may be a protective response due to the inflammatory milieu and the presence of non-removable autoantigens.

In conclusion, by analyzing a cohort of MPGN patients (Garam et al. [41]), a patient presenting both with FB- and FH-autoantibodies was found. We hypothesized that they could be involved in the disease pathomechanism and characterized in detail these concomitantly occurring autoantibodies. In contrast to the assumed pathogenic role, we found that in a variety of functional assays, these autoantibodies proved to be rather benign. Altogether, our data suggests that these autoantibodies are not only harmless but might be beneficial against the inflammatory microenvironment resulting from overwhelming complement activity in the kidney.

Though screening for autoantibodies against complement components in diseases is becoming more common, and more autoantibody—disease associations are described, a major limitation of these studies is the lack of functional investigations. It is, however, becoming increasingly clear that detection of complement autoantibodies does not necessarily indicate their pathogenicity. A limitation of our study is that it presents data on autoantibodies from only a single patient; however, together with previously reported data by others, it exemplifies the heterogeneity of FB- and FH-autoantibodies in MPGN. Our data also emphasizes that such autoantibodies may even have a protective functionality by themselves and are no causative factors in the disease. Thus, caution is warranted when assuming the disease-driving role of autoantibodies based solely on their detection. An epiphenomenal characteristic of such autoantibodies cannot be excluded. The role of anti-complement autoantibodies should be analyzed in larger patient cohorts as well as in individual cases to decipher the underlying pathological processes and disease mechanisms and further our understanding of the relevance of autoantibodies, as well as improve the diagnostic and therapeutic approaches in this and other diseases.

## Figures and Tables

**Table 1:** Characteristics and functional effects of the autoantibodies.

**Characterization**	**Titer**		1:3200 for both anti-FB and anti-FH
	**Isotype**		IgG2, IgG3, IgG4, IgGκ and IgGλ for both anti-FB and anti-FH
	**Binding site**		FB Ba and Bb; FH CCPs 1-4, 5-7, 15-20, 19-20, weak binding to CCPs 8-14; cross-reaction with FHR-1 and FHL-1
	**In vivo formed immune complex detection**		With both anti-FB and anti-FH
**Functional effects**	**Hemolysis**		Reduced when using whole IgG fraction
	**Solid-phase C3 convertase**	**Assembly**	No effect
		**Decay**	No effect *
		**Activity**	Decreased
	**Complement deposition**		No effect
	**C3b binding to FH**		Weak inhibition
	**FH cofactor activity**		No effect

* Enhanced convertase decay was measured when using the whole IgG fraction (containing FH-autoanti-FH IgG complexes).

## Data Availability

The raw data supporting the conclusions of this article will be made available by the authors upon request.

## Supplementary Materials

The following supporting information can be downloaded at https://www.mdpi.com/article/10.3390/biomedicines13030648/s1, Supplementary Table S1: Commercially obtained materials; Figure S1: Autoantibodies against FB and FH in the IC-MPGN patient; Figure S2: Autoantibody detection; Figure S3: Binding site and isotype of the autoantibodies; Figure S4: FB–autoanti-FB IgG complex determination; Figure S5: FH–autoanti-FH IgG complex determination; Figure S6: Cofactor activity of FH in the presence of autoantibodies.

## Abbreviations

The following abbreviations are used in this manuscript:

aHUS	atypical hemolytic uremic syndrome
AMD	age-related macular degeneration
AP	alternative pathway
C3G	C3 glomerulopathy
C3GN	C3 glomerulonephritis
C3Nef	C3 nephritic factor
C4Nef	C4 nephritic factor
C5Nef	C5 nephritic factor
CCP	complement control protein domain
CP	classical pathway
DDD	dense deposit disease
FB	factor B
FD	factor D
FH	factor H
FHL-1	factor H-like protein 1
FHR	factor H-related
FI	factor I
FP	factor P
GBM	glomerular basement membrane
GN	glomerulonephritis
IC-MPGN	immune complex-mediated membranoproliferative glomerulonephritis
MPGN	membranoproliferative glomerulonephritis
NHS	normal human serum
RRBC	rabbit red blood cell
