# Supplementary material for: Autoantibodies Against Factor B and Factor H Without Pathogenic Effects in a Patient with Immune Complex-Mediated Membranoproliferative Glomerulonephritis
Source: Biomedicines. 2025 Mar 6;13(3):648. doi: 10.3390/biomedicines13030648 (PMC11939916; doi:10.3390/biomedicines13030648)
Supplement: Supplementary file 1 [file biomedicines-13-00648-s001.zip › biomedicines-3495079-supplementary.pdf]

Supplementary Materials

Supplementary Table S1. Commercially obtained materials.

Material	Reference No.	Manufacturer
**Factor B (FB)**	#341262	Merck Life Science Kft. (Budapest, Hungary)
**C3b**	#204860	Merck Life Science Kft. (Budapest, Hungary)
**C3**	#204885	Merck Life Science Kft. (Budapest, Hungary)
**Factor I (FI)**	#341280	Merck Life Science Kft. (Budapest, Hungary)
**Factor H (FH)**	#341274	Merck Life Science Kft. (Budapest, Hungary)
**C1q**	#204876	Merck Life Science Kft. (Budapest, Hungary)
**Goat anti-FB antiserum**	#341272	Merck Life Science Kft. (Budapest, Hungary)
**Goat anti-FH antiserum**	#341276	Merck Life Science Kft. (Budapest, Hungary)
**Ba fragment of FB**	#A154	Complement Technologies, Inc. (Tyler, TX)
**Bb fragment of FB**	#A155	Complement Technologies, Inc. (Tyler, TX)
**Factor D (FD)**	#A409	Quidel (San Diego, CA)
**Properdin**	#A412	Quidel (San Diego, CA)
**Mouse monoclonal anti-FB (Bb)**	#A227	Quidel (San Diego, CA)
**Mouse monoclonal anti-FB (Ba)**	#A225	Quidel (San Diego, CA)
**Mouse monoclonal anti-C5b-9**	#A239	Quidel (San Diego, CA)
**C3a EIA kit**	#A032	Quidel (San Diego, CA)
**Complement active pooled normal human serum**	#A113	Quidel (San Diego, CA)
**Mouse monoclonal anti-FB (Bb)**	#HM2256	Hycult Biotech (Uden, The Netherlands)
**Human serum albumin (HSA)**	#A3782	Merck Life Science Kft. (Budapest, Hungary)
**Human alpha-1 antitrypsin (A1AT)**	#SRP6312	Merck Life Science Kft. (Budapest, Hungary)
**Purified human IgG**	#I2511	Merck Life Science Kft. (Budapest, Hungary)
**Goat anti-C1q**	#234390	Merck Life Science Kft. (Budapest, Hungary)
**Anti-human IgG**	#A6029	Merck Life Science Kft. (Budapest, Hungary)
**Anti-human IgM**	#A0420	Merck Life Science Kft. (Budapest, Hungary)
**Anti-human IgA**	#A0295	Merck Life Science Kft. (Budapest, Hungary)
**Mouse monoclonal anti-IgG1, clone HP-6001**	#I9388	Merck Life Science Kft. (Budapest, Hungary)
**Mouse monoclonal anti-IgG2, clone HP-6002**	#I9513	Merck Life Science Kft. (Budapest, Hungary)
**Mouse monoclonal anti-IgG3, clone HP-6050**	#I7260	Merck Life Science Kft. (Budapest, Hungary)
**Mouse monoclonal anti-IgGκ, clone KP-53**	#K4377	Merck Life Science Kft. (Budapest, Hungary)
**Mouse monoclonal anti-IgGλ, clone HP-6054**	#L6522	Merck Life Science Kft. (Budapest, Hungary)
**Lipopolysaccharide (LPS)**	#L4524	Merck Life Science Kft. (Budapest, Hungary)
**Mouse monoclonal anti-FH, clone C18/3**	#BPD-GAU-018-03-02	Enzo Life Sciences (Farmingdale, NY)
**Mouse anti-IgG4 HRP**	#9200-05	Southern Biotech (Birmingham, AL)
**Goat anti-human-IgG HRP**	#2014-05	Southern Biotech (Birmingham, AL)
**Mouse monoclonal IgG1, clone MOPC-21**	#BZ-400101	BioLegend (San Diego, CA)
**Goat anti-C3 F(ab')2**	#55062	MP Biomedicals (Solon, OH)
**Goat anti-C3 F(ab')2 HRP**	#55237	MP Biomedicals (Solon, OH)
**Rabbit anti-C3c**	#A0062	DAKO (Hamburg, Germany)
**Goat anti-mouse Ig HRP**	#P0447	DAKO (Hamburg, Germany)
**Rabbit anti-goat Ig HRP**	#P0449	DAKO (Hamburg, Germany)
**Swine anti-rabbit Ig HRP**	#P0217	DAKO (Hamburg, Germany)
**Rabbit red blood cells (RRBCs)**		Culex Bt. (Budapest, Hungary)

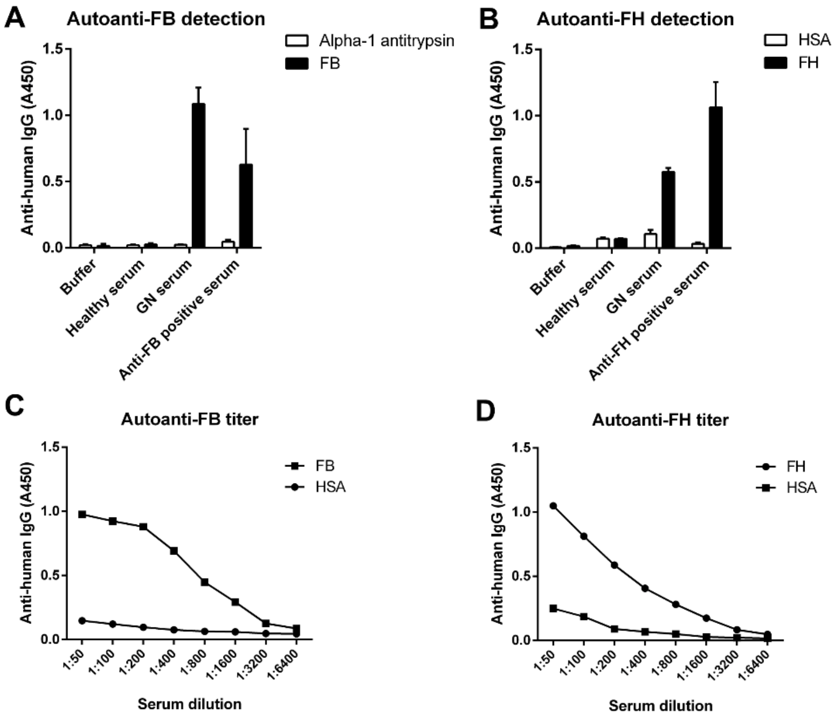

Supplementary Figure S1. Autoantibodies against FB and FH in the IC-MPGN patient. Autoanti-FB (**A**) and autoanti-FH (**B**) were detected in the serum of the patient compared to healthy serum in ELISA. Serum from a DDD patient was used as autoanti-FB positive control and serum from an atypical HUS patient was used as autoanti-FH positive control. Data are mean ± SD from three and two independent experiments, respectively. The titer was determined using serial dilutions of the serum of the patient on immobilized FB (**C**) and FH (**D**) and binding was compared to HSA. FB: factor B, FH: factor H, DDD: dense deposit disease, HUS: hemolytic uremic syndrome, HSA: human serum albumin

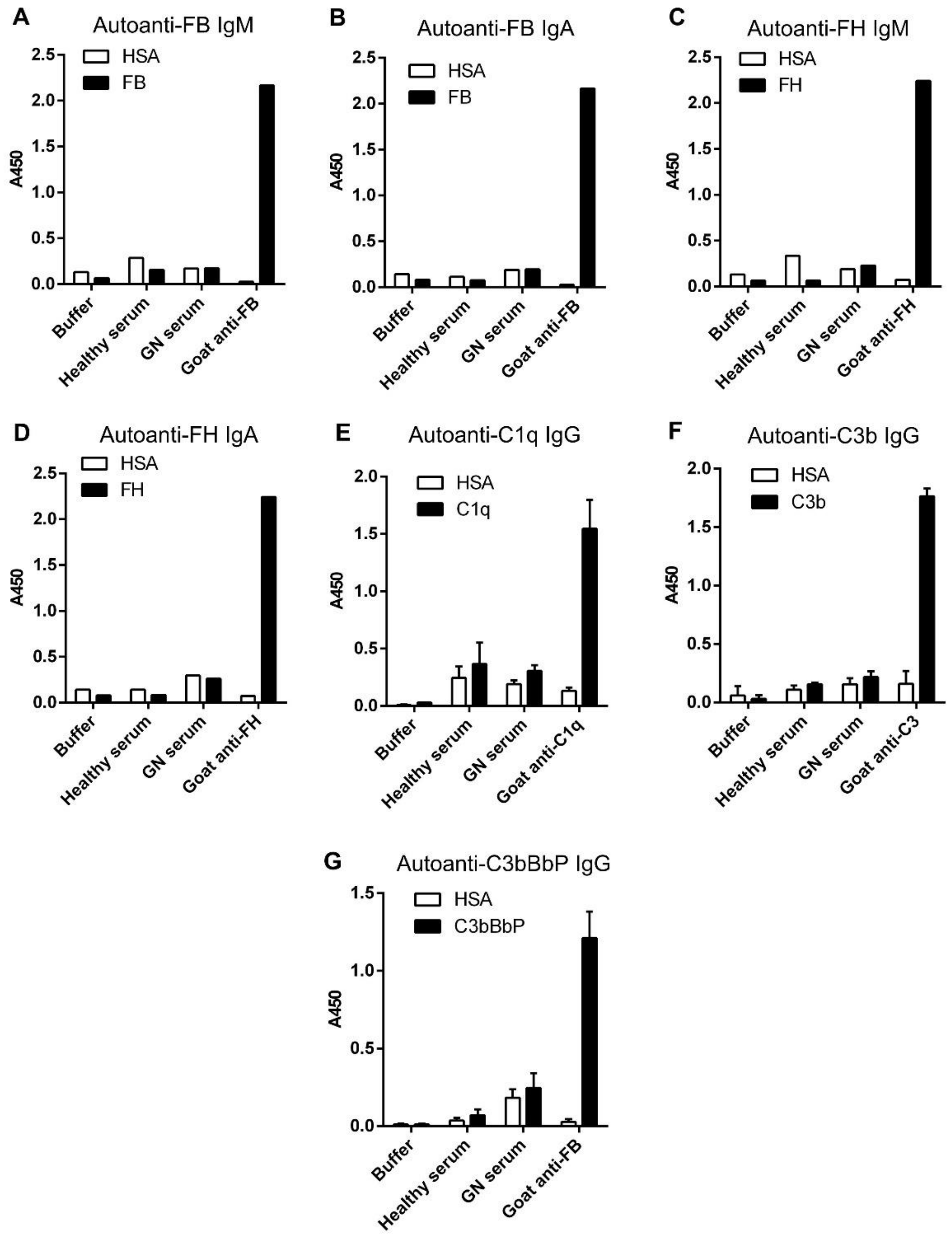

Supplementary Figure S2. Autoantibody detection. Anti-FB IgM (**A**) and IgA (**B**), anti-FH IgM (**C**) and IgA (**D**), anti-C1q IgG (**E**), anti-C3b IgG (**F**) and anti-C3bBbP IgG (**G**) were not detected in the serum of the patient. Healthy serum was used as negative control. Goat anti-FB, anti-FH, anti-C1q and anti-C3 were positive controls. Panels (**A**) - (**D**) show the result of single experiments, (**E**) and (**F**) show the mean ± SD of two experiments, (**G**) represents the mean ± SD of three independent experiments.

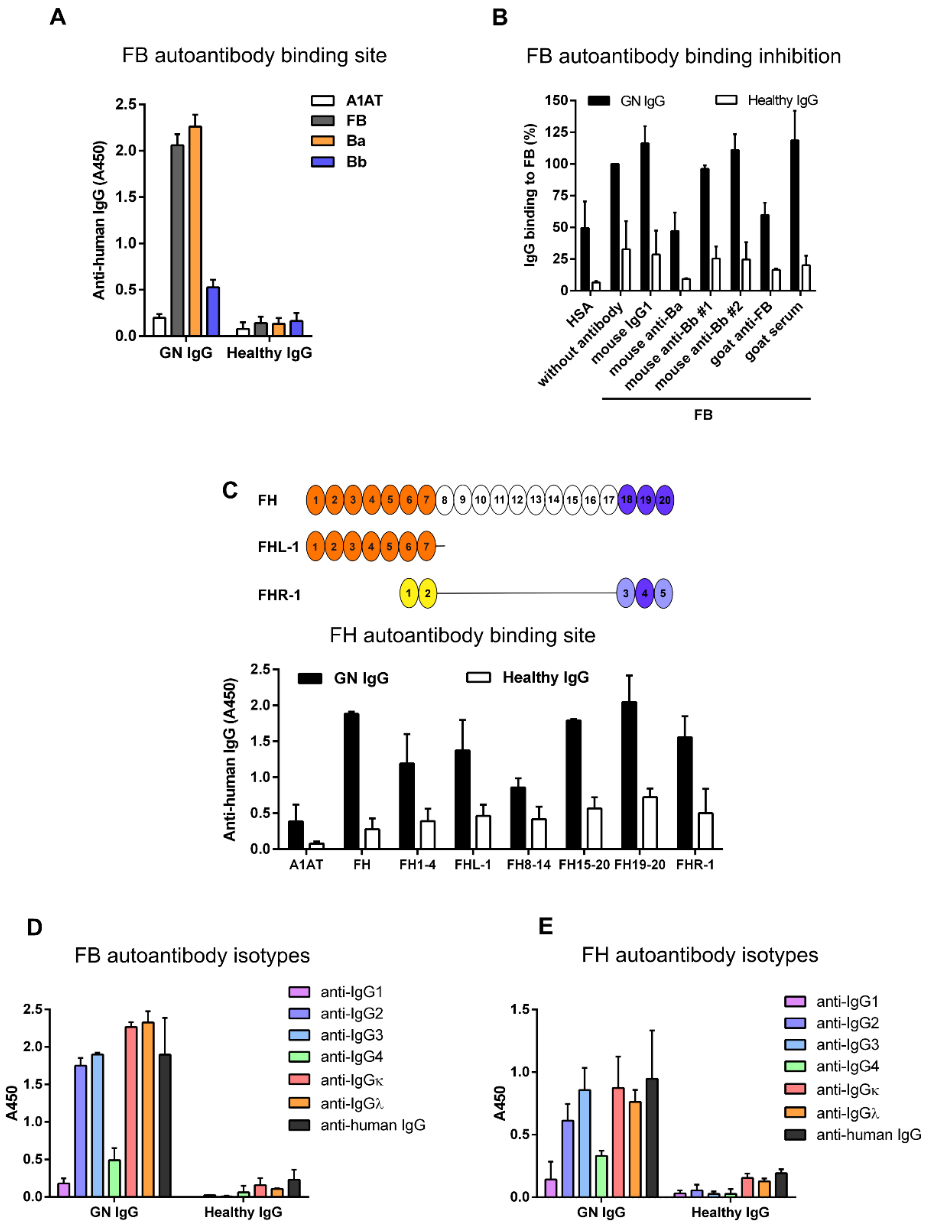

Supplementary Figure S3. Binding site and isotype of the autoantibodies. (**A**) FB autoantibody binding site was determined with FB fragments Ba and Bb. Autoantibodies bound to full-length FB, Ba and Bb fragment. (**B**) FB autoantibody binding to FB was inhibited by monoclonal anti-Ba antibody and polyclonal anti-FB. (**C**) The binding sites of the anti-FH autoantibodies are localized on the N-terminal part of FH (FH1-4) as well as on the C-terminal part (FH15-20, FH19-20) and a weak binding could be detected to the middle region (FH8-14); the autoantibodies also cross-react with FHL-1 and FHR-1. Domain composition of FH, FHL-1 and FHR-1 proteins arranged by homology is shown above the graph. Identical colors and color intensity indicate the degree of structural similarity. (**D**) Autoanti-FB and (**E**) autoanti-FH isotypes are IgG2, IgG3, IgG4, IgGκ and IgGλ. Values obtained with buffer control as a background were subtracted from the values obtained from the healthy control and HUN593 patient before plotting. Data are means ± SD from two independent

experiments. FB: factor B, FH: factor H, FHL: factor H-like, FHR: factor H-related, GN: glomerulonephritis

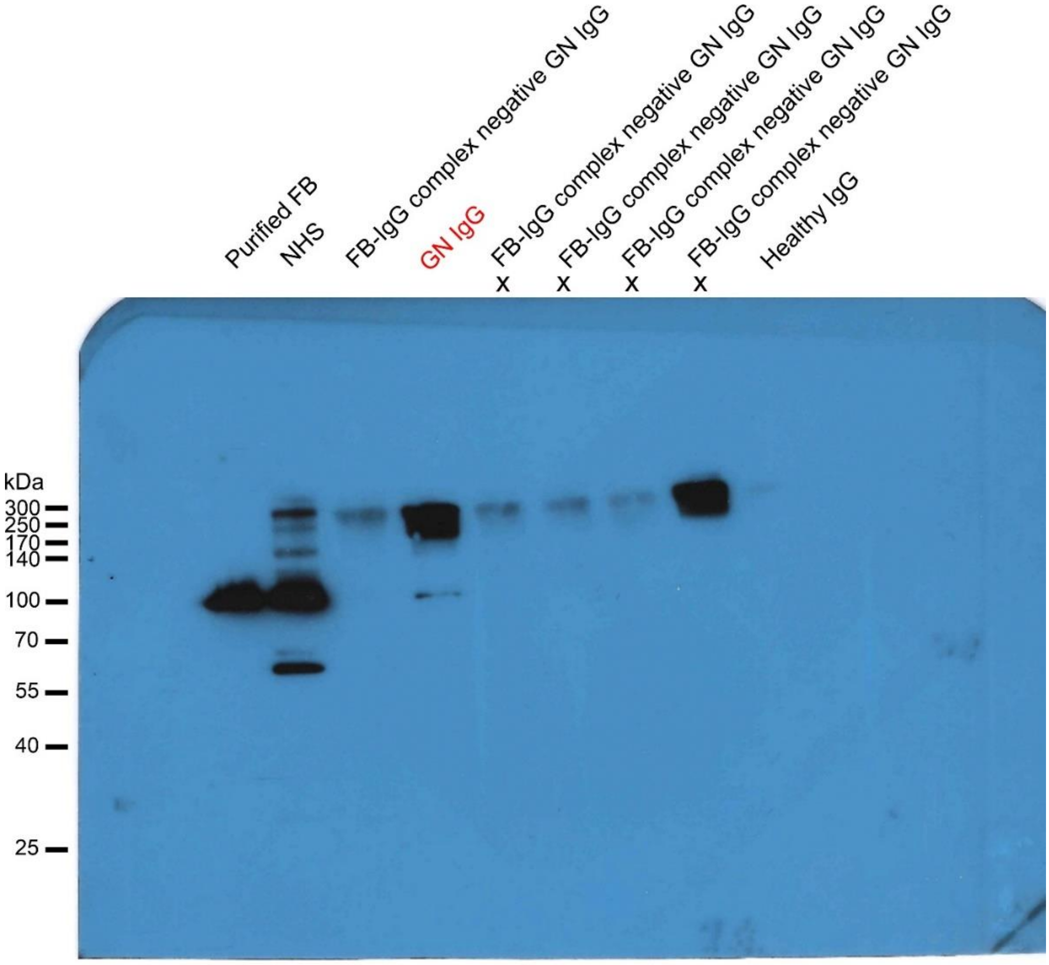

Supplementary Figure S4. FB–autoanti-FB IgG complex determination. FB–anti-FB complexes were detected in the IgG of the analyzed patient (GN IgG), but not in the healthy control sample, or in IgG of other patients with GN. This is the unprocessed film for Figure 2A. X indicates the lanes not included in the final figure. FB: factor B, NHS: normal human serum, GN: glomerulonephritis

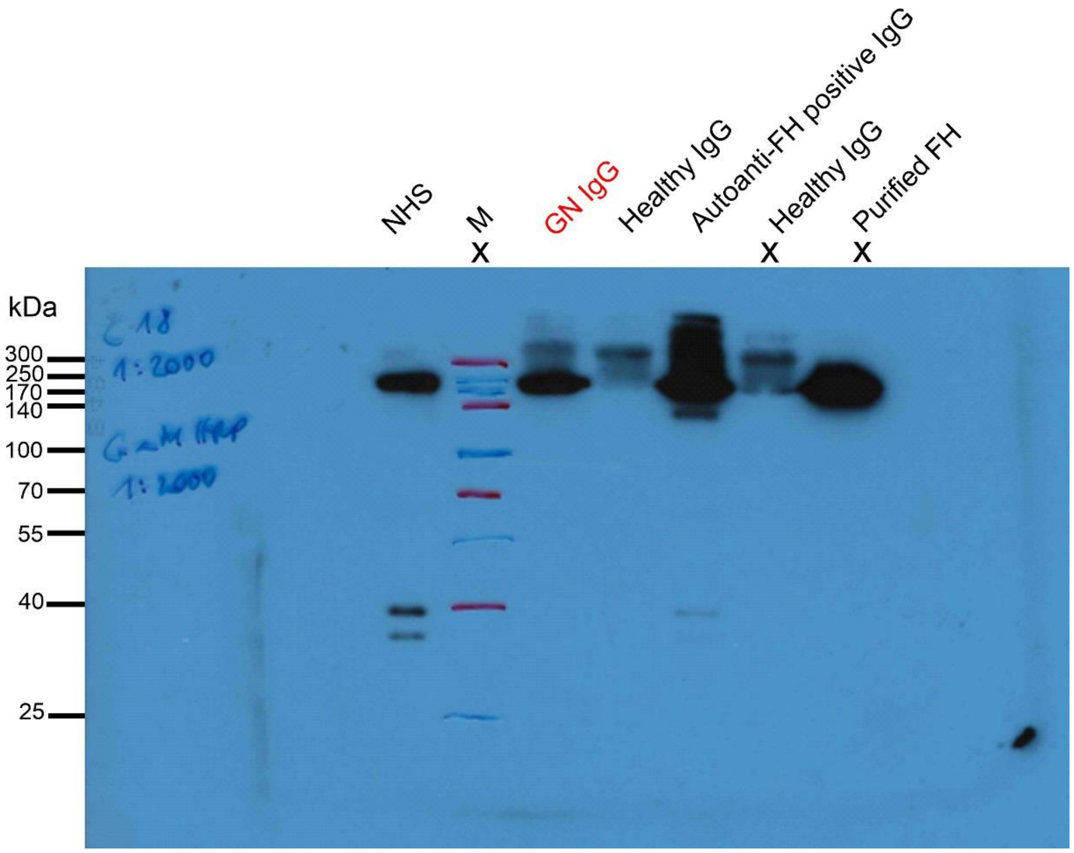

Supplementary Figure S5. FH–autoanti-FH IgG complex determination. FH–autoanti-FH IgG complexes were detected in the patient's IgG (GN IgG). FH was not present in the healthy control IgG samples. IgG of a FH-autoantibody positive aHUS patient was loaded as positive control, containing complexes both with FH and FHR-1 and normal human serum was run as technical control. The antibody used for the detection (mouse anti-FH, (clone: C18/3) recognizes both FH and FHR-1). This is the unprocessed film for Figure 2B. X indicates the lanes not included in the final figure. NHS: normal human serum, GN: glomerulonephritis, FH: factor H

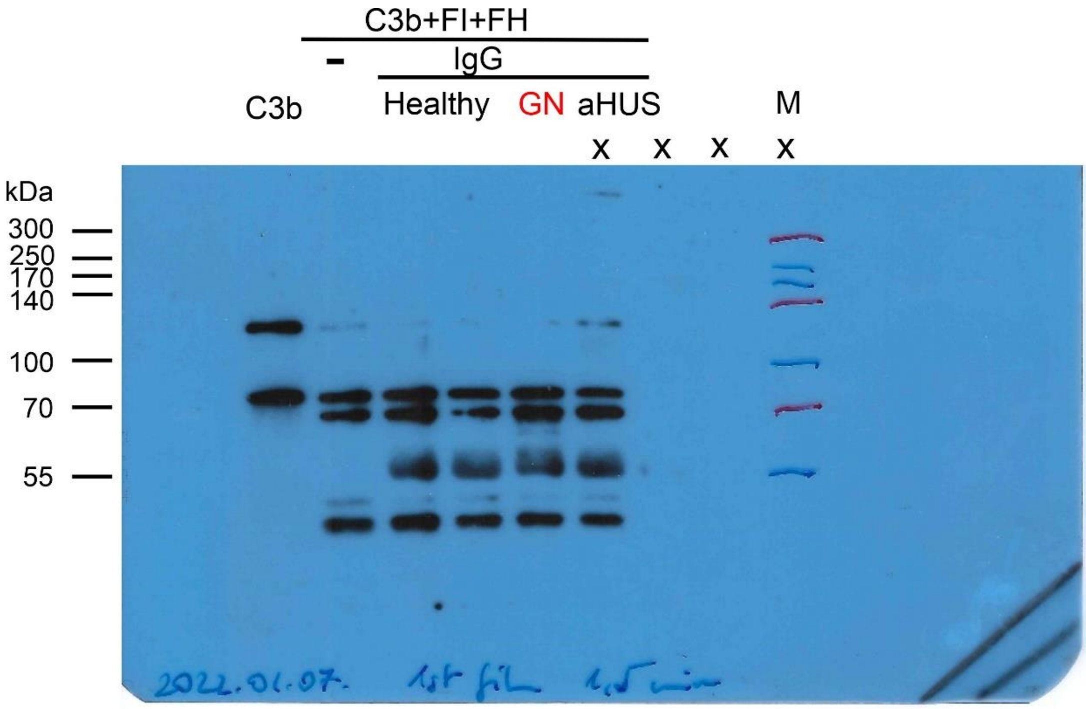

Supplementary Figure S6. Cofactor activity of FH in the presence of autoantibodies. Autoantibodies of the GN patient (lane 5) did not influence the cofactor activity of FH. In addition to the GN IgG, two healthy IgG (lanes 3-4) samples and an autoanti-FH positive aHUS IgG (lane 6) were used as controls. C3b alone and C3b with FI and FH were run as technical controls (lanes 1-2). This is the unprocessed film for Figure 4D. X indicates the lanes not included in the final figure. FI: factor I, FH: factor H, GN: glomerulonephritis, aHUS: atypical hemolytic uremic syndrome
